# Safety and Incidence of Cardiovascular Events in Chinese Patients with Acute Coronary Syndrome Treated with Ticagrelor: the 12-Month, Phase IV, Multicenter, Single-Arm DAYU Study

**DOI:** 10.1007/s10557-018-6772-3

**Published:** 2018-02-28

**Authors:** Runlin Gao, Yongjian Wu, Hengliang Liu, Guohai Su, Zuyi Yuan, Aidong Zhang, Yong Wang, Zhirong Wang, Yan Wang, Huanyi Zhang, Yang Zheng, Lei Liu, Lijun Shen, Maria Leonsson-Zachrisson, Yaling Han, on behalf of the DAYU study investigators, Wei Miao, Guoying Geng, Wenjie Han, Lei Wu

**Affiliations:** 1 Fuwai Hospital, National Center for Cardiovascular Diseases, Chinese Academy of Medical Sciences, 167 Beilishi Road, Xi Cheng District, Beijing, China; 2 Zhengzhou People Hospital, Zhengzhou, China; 3 Jinan Central Hospital, Jinan, China; 4 First Affiliated Hospital of Medical College of Xi’An Jiaotong University, Xi’an, China; 5 The First Affiliated Hospital of Jinan University, Guangzhou, China; 6 China-Japan Friendship Hospital, Beijing, China; 7 The Affiliated Hospital of Xuzhou Medical University, Xuzhou, China; 8 Xiamen Heart Center, Xiamen, China; 9 The Central Hospital of Tai’an, Tai’an, China; 10 The First Hospital of Jilin University, Changchun, China; 11 AstraZeneca China, Shanghai, China; 12 AstraZeneca R&D, Mölndal, Sweden; 13 The General Hospital of Shenyang Military Region, Shenyang, China

**Keywords:** Acute coronary syndrome, Chinese patients, Ticagrelor, Safety

## Abstract

**Purpose:**

Ticagrelor is an orally administered, reversibly binding, direct-acting P2Y_12_ receptor antagonist previously evaluated in several phase III trials. This phase IV, multicenter, single-arm trial assessed the safety and incidence of cardiovascular (CV) events with ticagrelor in Chinese patients experiencing an acute coronary syndrome (ACS).

**Methods:**

Patients hospitalized with an ACS received ticagrelor (180 mg loading dose, 90 mg twice daily thereafter) plus low-dose aspirin (75–100 mg/day) for up to 12 months. Safety was evaluated via PLATO-defined bleeding events, adverse events (AEs), serious AEs, and laboratory measurements. The incidence of major CV events was also evaluated.

**Results:**

The safety population included 2001 patients. During ticagrelor treatment, 426 (21.3%) patients had at least one PLATO-defined bleeding AE, mainly minimal bleedings (*n* = 333). Major bleeding events occurred in 27 (1.3%) patients, including fatal/life-threatening bleeding in 17 (0.8%) patients and other major bleeding in 11 (0.5%) patients, with a Kaplan-Meier estimate of patients with the event (95% CI) of 1.6% (1.1–2.3%). In total, 784 (39.2%) patients had at least one non-bleeding AE, the majority of which were mild in severity. The composite endpoint of CV death, myocardial infarction, and stroke occurred in 83 (4.1%) patients.

**Conclusions:**

Ticagrelor plus low-dose aspirin for up to 1 year was associated with a low rate of major bleeding events and a low incidence of major CV events (CV death, myocardial infarction, stroke) in Chinese patients with ACS. The overall safety profile of ticagrelor in this population was in line with current prescribing information.

**Electronic supplementary material:**

The online version of this article (10.1007/s10557-018-6772-3) contains supplementary material, which is available to authorized users.

## Introduction

Currently, ~ 230 million individuals have cardiovascular disease (CVD) in China [1]. Chinese patients experiencing an acute coronary syndrome (ACS)—encompassing unstable angina, non-ST elevation myocardial infarction (NSTEMI), and ST elevation myocardial infarction (STEMI)—have a high mortality rate [2, 3]. Thus, improved use of guideline-recommended therapies in this population is required [4]. Dual antiplatelet therapy (DAPT) with aspirin and a P2Y_12_ receptor inhibitor (ticagrelor, clopidogrel or prasugrel for ≥ 12 months) represents the cornerstone of ACS management [5–9].

Ticagrelor, an orally administered, direct-acting, reversibly binding P2Y_12_ receptor antagonist, inhibits adenosine diphosphate-induced platelet aggregation [10, 11] and cellular uptake of adenosine via inhibition of the equilibrative nucleoside transporter 1 [12]. Ticagrelor with low-dose aspirin is approved in more than 100 countries (including China) to reduce the rate of CV death, myocardial infarction (MI), and stroke in ACS patients (180 mg loading dose then 90 mg twice daily [b.i.d] for the first year post-ACS event) [13]. In some countries and regions (e.g., USA, Japan, Hong Kong, and EU countries), ticagrelor is approved to reduce the rate of CV death, MI, and stroke in patients with a history of MI (60 mg b.i.d from 1 year post-MI).

The efficacy and safety of ticagrelor have been established in two, key phase III trials, PLATO in patients with ACS [14] and PEGASUS-TIMI 54 in patients with a prior MI [15]. In PLATO, ticagrelor plus aspirin significantly reduced the rate of the primary composite endpoint (MI, stroke, and death from vascular causes) in ACS patients versus clopidogrel plus aspirin [14]. Moreover, the hazard ratio (HR) (95% confidence interval [CI]) for cardiovascular death in PLATO was 0.79 (0.69–0.91; *p* = 0.001) [14]. PLATO reported no difference in the overall rate of major bleeding between ticagrelor and clopidogrel. However, compared with clopidogrel, ticagrelor was associated with a higher rate of PLATO-defined major bleeding not related to coronary artery bypass grafting (CABG), including a higher incidence of fatal intracranial bleeding but fewer instances of non-intracranial types of fatal bleeding; overall fatal bleeding was not different between ticagrelor and clopidogrel [14].

PLATO and PEGASUS-TIMI 54 were conducted worldwide, and recruited some Asian patients [14, 15]. In PLATO, 587 ACS patients of Chinese ethnicity were enrolled (i.e., 3.1% of 18,758 enrolled patients) and 577 were randomized and received study treatment. Of these Chinese patients, 418 were enrolled and 416 were randomized in Mainland China [AstraZeneca data on file]. In a retrospective PLATO analysis, the efficacy and safety of ticagrelor versus clopidogrel in Asian patients (*n* = 1106) were similar to non-Asian patients (*n* = 17,515) [16]. However, clinical data with DAPT in Asian patients is limited, and Asian patients are hypothesized to have different risk profiles for thrombophilia and bleeding versus Caucasian patients [17]. Furthermore, clopidogrel requires metabolic activation via several cytochrome P450 (CYP) isoenzymes, particularly CYP2C19 [18]. CYP2C19 loss-of-function alleles result in clopidogrel resistance [19], and the prevalence of these alleles is high in Chinese patients (e.g., up to 60% [20–22]). Patients with CYP2C19 loss-of-function alleles treated with clopidogrel are at high risk of adverse cardiovascular outcomes [19, 21]. Thus, alternative drugs are of high interest in Chinese ACS patients needing alternative DAPT, such as ticagrelor [20, 22]. The mean bioavailability of ticagrelor and AR-C124910XX (the active metabolite of ticagrelor) is higher (~ 20–40%) in Asian subjects versus Caucasians, although this difference is unlikely to be clinically relevant [23–26]. Thus, further clinical data with ticagrelor at the approved dosing regimen in Chinese patients with ACS are required.

In agreement with the Chinese Food and Drug Administration (CFDA), the present DAYU study was conducted to further assess the safety profile of ticagrelor in a large population of Chinese patients with ACS. The primary objective was to describe the safety and tolerability of ticagrelor by assessing bleeding events and other serious adverse events (SAEs) during 1-year follow-up. The secondary objectives were to describe the incidence of CV events (including CV death, MI, stroke) with ticagrelor during 1-year follow-up in Chinese ACS patients, and to explore the incidence of fatal/life-threatening bleeding and major bleeding in various patient subgroups.

## Methods

### Study Design

The DAYU study (NCT01870921) was an interventional, open-label, multicenter, single-arm, phase IV trial conducted in China. Patients were enrolled on the first day of hospitalization following the index ACS event. The treatment approach for the index ACS event (i.e., invasive therapy or medical management) was at the discretion of the local investigator. Patients were also treated with ticagrelor (180 mg loading dose followed by 90 mg b.i.d), plus low-dose aspirin (75–100 mg/day), for 12 months. Given that the ACS patients were in a critical condition and in keeping with current guidelines for clinical management of ACS, initiation of the study drug (ticagrelor) took place as soon as possible after randomization and was not delayed to allow procedures (including laboratory testing and angiograms) to be conducted. Ticagrelor was supplied to patients, who returned all unused study medication/empty packages at each visit for assessment of treatment compliance by pill count.

The study was conducted in accordance with the Declaration of Helsinki and the International Conference on Harmonization/Good Clinical Practice (GCP) Guidelines, and followed applicable regulatory requirements including AstraZeneca’s policy on bioethics. The local Institutional Review Boards or Independent Ethics Committees approved the final protocol and amendment. Of the 104 sites that participated in the DAYU study, 93 (89.4%) were CFDA-GCP certified clinical trial institutions. Such institutions obtain certification from the state CFDA prior to conducting clinical trials. The CFDA monitors site personnel and corresponding facilities to ensure that the institution is appropriate for conducting clinical trials on drugs for human use. After completion of the study, the study operation team conducted the self-verification process at 84 sites and for 1575 patients, as per the CFDA guidance (i.e., prior to on-site inspection requirements introduced in 2016). Quality-control approval and certified site summaries were received from all the 104 participating investigator sites. There were no external inspections of the study sites or data.

### Patients

Eligible patients were ethnic Chinese men and women (non-pregnant, or post-menopausal, or surgically sterile) aged ≥ 18 years, with an index ACS event of NSTEMI, STEMI, or unstable angina. Qualifying events met the following criteria: hospitalization with cardiac ischemic symptoms for ≥ 10 min at rest and either (1) persistent ST segment elevation ≥ 1 mm (0.1 mV) in two or more contiguous leads and primary percutaneous coronary intervention (PCI) planned, or (2) new or presumed new left bundle branch block and primary PCI planned, or (3) ST segment changes on electrocardiogram (ECG) indicative of ischemia and/or positive biomarker evidence of myocardial necrosis.

Major exclusion criteria reflected contraindications and precautions detailed in the ticagrelor prescribing information and included contraindications to ticagrelor (hypersensitivity, active bleeding, moderate or severe liver disease, history of intracranial bleed, major surgery within 30 days); planned urgent CABG within 7 days from enrolment; use of non-selective, non-steroidal anti-inflammatory drugs that could not be stopped; or oral anticoagulation therapy within 30 days of enrolment or that could not be stopped. Concomitant therapy with strong CYP 3A inhibitors, CYP3A substrates with narrow therapeutic indices, or strong CYP3A inducers was prohibited.

In addition to fulfilling eligibility criteria, all patients were required to accept study participation in the acute situation by providing written informed consent before any study-related procedures were initiated.

### Safety Assessments

Patients returned to the clinic at 6 weeks, 3, 6, 9, and 12 months after start of study drug for assessments of adverse events (AEs) and concomitant medication. AEs were also collected in medical records, and these records were checked during the study clinic visits and during the monitoring visits as well as during site self-inspection. In addition, physical examinations, vital signs and safety laboratory measurements were included at 6 weeks and at 12 months (or at the end of treatment, if earlier). Laboratory assessments could be conducted at any visit at the discretion of the investigator. A follow-up visit was scheduled 2 weeks after the end of treatment.

AEs and SAEs, including bleeding, were recorded by the local investigators according to standard classifications used in clinical studies. These events were classified by the investigators as mild (i.e., awareness of sign or symptom, which is easily tolerated), moderate (i.e., discomfort sufficient to cause interference with normal activities), or severe (i.e., incapacitating, and inability to perform normal activities). All bleeding events were also categorized by the local investigators according to the definitions used in the PLATO study [14, 27] (Supplementary methods of Supplementary Material Online).

### Cardiovascular Events

Clinical endpoints were evaluated by local investigators and included CV death (death from CV or cerebrovascular causes), MI (elevation of myocardial necrosis biomarkers with ≥ 1 of the following: recurrent cardiac ischemic symptoms, development of new pathological Q waves in the ECG, and new or presumed new ECG changes indicative of ischemia), and stroke (a neurological deficit caused by an ischemic or hemorrhagic central nervous system event with residual symptoms for ≥ 24 h after onset or leading to death). Ischemic CV events were not reported as AEs.

### Sample Size and Data Analyses

In PLATO, the risk of fatal/life-threatening bleeding in the Chinese subgroup was 5.7% [AstraZeneca data on file] and similar to the risk of 5.8% in the full cohort [14]. Thus, 2000 patients would be required for the rate of fatal/life-threatening bleeding to be estimated with a precision of 1% (95% CI) in the current study.

A patient was considered to be compliant with ticagrelor treatment if > 80 and < 120% of the planned number of tablets were taken during their treatment period (excluding any days when the study drug was temporarily stopped). Safety data were summarized descriptively for the safety population. Frequency and percentage of patients in each category for PLATO-defined bleeding events, AEs, SAEs, and AEs of special interest (dyspnea and hyperuricemia) were tabulated for events reported during ticagrelor treatment, unless otherwise indicated. An exploratory analysis of fatal/life-threatening bleeding events was conducted for the following subgroups: sex (males vs. females), age (< 75 years vs. ≥ 75 years), use of glycoprotein (GP)IIb/IIIa inhibitors (yes vs. no), and treatment (medically managed vs. invasive therapy). For time-to-major CV event (composite of CV death/MI/stroke), Kaplan-Meier (KM) plots were constructed, and the KM estimate and 95% CI for this endpoint, and the individual components at 12 months (365 days), were calculated.

## Results

### Patients and Treatment

The DAYU study was conducted across 104 investigational centers in 21 provinces or municipalities of China (Participating sites in Supplementary Material Online) between 26 June 2013 (first patient enrolled) and 15 September 2015 (last patient completed the study).

**Fig. 1:**
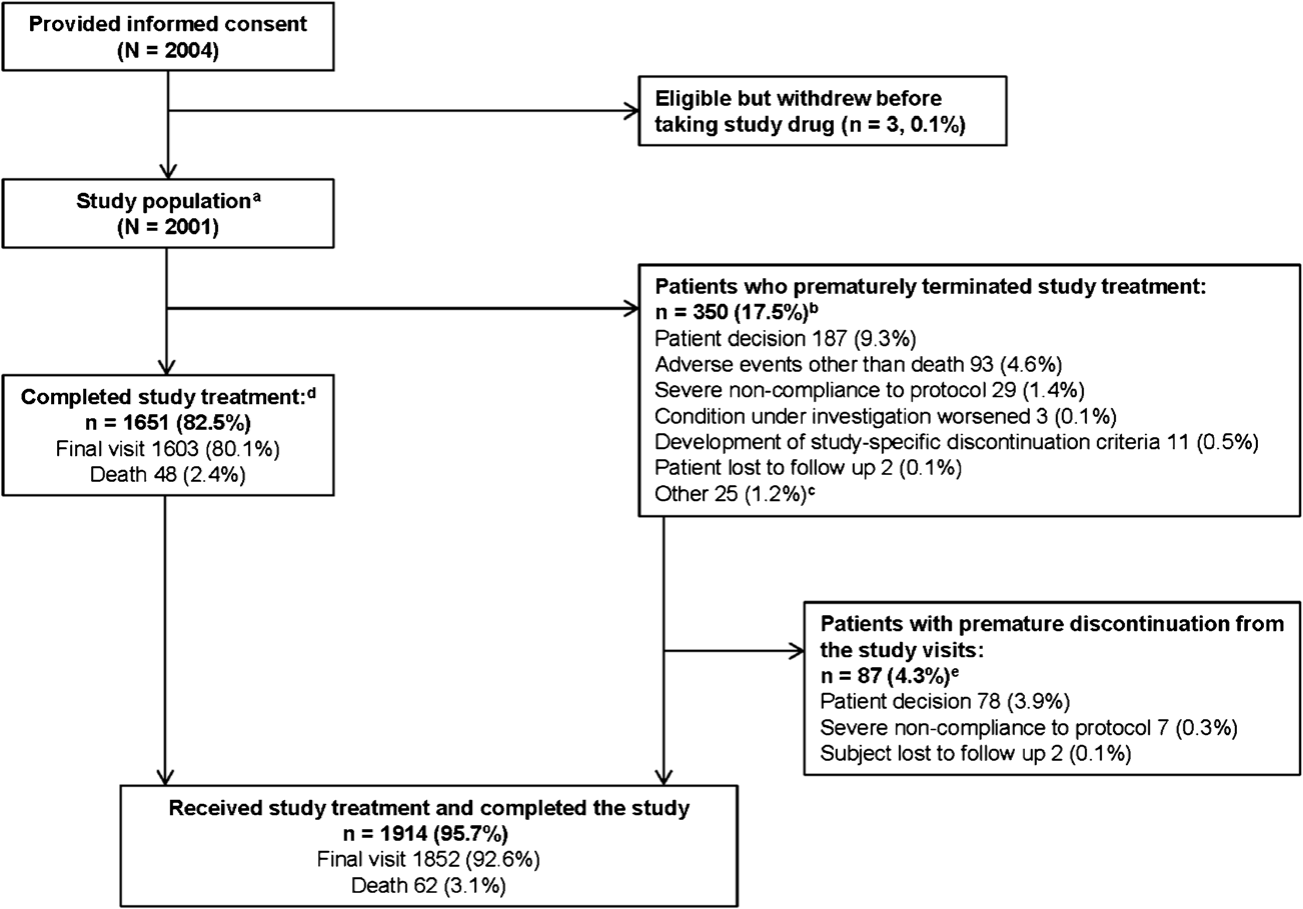
Patient disposition. **a** Sixty-two patients failed screening as they did not meet inclusion criteria and received study medication. These patients are included in the safety analyses. **b** For patients who prematurely discontinued the study drug, the study visits continued, if these were acceptable to the patient. **c** Other reasons for early study termination included: enrollment error (*n* = 6), planned/conducted coronary artery bypass grafting (*n* = 5), angiogram results indicated no need for antiplatelet therapy (*n* = 5), patient withdrew consent (*n* = 3), investigator decision (*n* = 3), reduced bleeding risk (*n* = 1), imprisonment (*n* = 1), and car accident (*n* = 1). **d** Completion of study treatment was defined as treatment continuation up to the final visit or death, whichever occurred first. **e** Completion of study visits was defined as completion of the visits up to the final visit or death, whichever occurred first

Overall, 2004 patients provided written, informed consent, 2001 received ticagrelor and constituted the safety population (Fig. 1). Of these patients, 1939 were eligible for study inclusion. The remaining 62 patients (3% of safety population) received ticagrelor despite not fulfilling eligibility criteria, most commonly due to the final diagnosis of the index event not meeting study STEMI or NSTEMI criteria. Consistent with current medical guidelines on antiplatelet therapies, the study protocol required patients to be enrolled as quickly as possible after presentation in order to maximize potential clinical benefits of treatment. Therefore, in these cases, it is likely that as medical assessment continued and more information became available, the investigator made relevant adjustments to the final diagnosis. A total of 1651 patients (82.5% of safety population) completed study treatment (i.e., patients who continued ticagrelor for 12 months or until death), while 350 patients of the safety population prematurely terminated treatment, primarily due to patient decision (9.3%), AEs (4.6%), and non-compliance (1.4%). Of the 29 patients who discontinued the study due to severe non-compliance to the protocol, six patients had nine AEs reported during the last month prior to discontinuation, no SAEs were reported. The nine reported AEs were anemia, diarrhea, non-cardiac chest pain, epistaxis, blurred vision, nausea, vomiting, abdominal pain, and urticaria. None of these AEs resulted in treatment discontinuation in these patients. Of the patients who prematurely terminated study treatment, 67 patients were given clopidogrel as an alternative antiplatelet agent (no other antiplatelet agents were used post-discontinuation).

Table 1 shows the baseline characteristics of the safety population. Overall, 1999 (99.9%) patients had at least one concomitant medication, most commonly aspirin (99.2%), HMG-CoA reductase inhibitors (95.3%), or heparin (81.4%).

**Table 1:** Patient demographics and disease characteristics (safety population)

Characteristic	Number (%) of patients, unless otherwise stated (*n* = 2001)
Age, mean (range), years	59.3 (25–96)
Age group	
< 75 years	1854 (92.7)
≥ 75 years	147 (7.3)
Sex	
Male	1579 (79)
Female	422 (21)
BMI, mean (SD), kg/m^2^	24.8 (3.3)
Race	
Chinese	2001 (100)
Medical history	
Hypertension	1102 (55.1)
Coronary artery disease	686 (34.3)
Angina pectoris	656 (32.8)
Diabetes	520 (26.0)
Dyslipidemia	380 (19.0)
Peptic ulcer disease	96 (4.8)
Gout	29 (1.4)
Chronic obstructive pulmonary disease	19 (0.9)
Final diagnosis of index event	
Unstable angina pectoris	488 (24.4)
ST elevation MI	1064 (53.2)
Non-ST elevation MI	424 (21.2)
Other	25 (1.2)
Use of GPIIb/IIIa inhibitors prior to first ticagrelor dose	30 (1.5)
Treatment approach at enrolment	
Invasive therapy	1756 (87.8)
Medically managed therapy	245 (12.2)

*BMI* body mass index, *MI* myocardial infarction, *SD* standard deviation

Mean (standard deviation [SD]) treatment compliance was 98.5% (9%). Mean (SD) exposure to ticagrelor was 309 (126) days. Overall, 82.6 and 81.2% of patients had > 6 or > 9 months of exposure, respectively. Some patients received ticagrelor treatment for > 12 months; 13.4% received ticagrelor for > 12–≤ 13 months, 0.4% for > 13–≤ 14 months, and 0.1% for > 14 months.

### Bleeding AEs

Overall, of the safety population, 426 (21.3%) patients experienced at least one bleeding AE (see the next section for bleeding events classified according to PLATO definition) (Table 2), and 422 (21.1%) had bleeding AEs during treatment. On-treatment bleeding AEs were mild (17.0%), moderate (2.4%), or severe (1.7%). Fifty-four (2.7%) patients experienced at least one bleeding SAE. Seven, 15, and 32 patients experienced mild, moderate, or severe bleeding SAEs, respectively, during treatment. On-treatment, bleeding AEs necessitated ticagrelor discontinuation in 55 (2.7%) patients. Treatment-related bleeding AEs (per investigator judgment) were reported in 294 (14.7%) patients; 41 (2.0%) patients had treatment-related SAEs.

**Table 2:** Bleeding adverse events (overall incidence ≥ 0.2%) by causality and preferred term (safety population)

Preferred term	Ticagrelor 90 mg b.i.d *n* = 2001		
	Related to treatment *n* (%)	Not related to treatment *n* (%)	Overall *n* (%)
Patients with at least one bleeding AE	296 (14.8)^a^	130 (6.5)	426 (21.3)
Ecchymosis	83 (4.1)	27 (1.3)	110 (5.5)
Gingival bleeding	68 (3.4)	23 (1.1)	91 (4.5)
Epistaxis	46 (2.3)	32 (1.6)	78 (3.9)
Subcutaneous hemorrhage	24 (1.2)	16 (0.8)	40 (2.0)
Upper gastrointestinal hemorrhage	18 (0.9)	4 (0.2)	22 (1.1)
Gastrointestinal hemorrhage	12 (0.6)	7 (0.3)	19 (0.9)
Hemoptysis	10 (0.5)	5 (0.2)	15 (0.7)
Petechiae	10 (0.5)	2 (0.1)	12 (0.6)
Hematuria	6 (0.3)	4 (0.2)	10 (0.5)
Hematochezia	5 (0.2)	3 (0.1)	8 (0.4)
Contusion	6 (0.3)	1 (0.0)	7 (0.3)
Occult blood positive	3 (0.1)	3 (0.1)	6 (0.3)
Conjunctival hemorrhage	4 (0.2)	1 (0.0)	5 (0.2)
Cerebral hemorrhage	3 (0.1)	1 (0.0)	4 (0.2)
Hemorrhoidal hemorrhage	3 (0.1)	1 (0.0)	4 (0.2)
Retinal hemorrhage	2 (0.1)	2 (0.1)	4 (0.2)
Urethral hemorrhage	3 (0.1)	1 (0.0)	4 (0.2)

^a^Causality assessment as judged by the investigator. One patient had an AE of epistaxis before start of study treatment which was reported as related to the study drug as this patient was prescribed ticagrelor prior to enrolment. This summary table includes events regardless if the onset was during or after treatment with ticagrelor

*AE* adverse event, *b.i.d* twice daily

### Bleeding Events Classified According to PLATO Definition

PLATO-defined bleeding events during and post-treatment combined are shown in Table 3. PLATO-defined major, minor, and minimal bleedings (composite) occurred in 426 (21.3%) patients, the majority of whom (*n* = 333) had minimal bleeding only.

**Table 3:** PLATO-defined bleeding events by severity (safety population)

Bleed severity	Ticagrelor 90 mg b.i.d (*n* = 2001)	
	Patients with bleeding *n* (%)	Number of bleeding events
Total major bleeding	27 (1.3)	28
Life-threatening/fatal	17 (0.8)	17
Fatal	4 (0.2)	4
Life-threatening	13 (0.6)	13
Major, other	11 (0.5)	11
Composite of major and minor bleeding	93 (4.6)	106
Minor bleeding	66 (3.3)	78
Composite of major, minor, and minimal bleeding	426 (21.3)	640
Minimal bleeding	353 (17.6)	534

This summary table includes all recorded events regardless of timing relative to study treatment

*b.i.d* twice daily

Among PLATO-defined major bleeding events, fatal/life-threatening bleeding was observed in 17 (0.8%) patients, with a KM estimated event risk (95% CI) of 1.0% (0.6–1.6). Fourteen patients experienced fatal/life-threatening spontaneous bleeding and three patients had traumatic bleeding; the two most common bleeding sites were gastrointestinal tract (*n* = 10) and intracranial system (*n* = 4). Overall, four of these events were fatal and none were procedurally related. Other major bleeding events were reported in 11 (0.55%) patients, with one patient experiencing both fatal/life-threatening and other major bleeding events. The KM estimated event risk (95% CI) for the 27 patients who experienced fatal/life-threatening bleeding and/or other major bleeding was 1.6% (1.1–2.3).

Minor bleeding occurred in 66 (3.3%) patients, with a KM estimated event risk (95% CI) of 3.7% (2.9–4.7). For the 93 patients with major or minor bleeding, the KM estimated event risk (95% CI) was 5.2% (4.3–6.4).

### Exploratory Analyses of PLATO-Defined Bleeding Events

Exploratory analyses of PLATO-defined bleeding events by patient subgroup showed that all 17 fatal/life-threatening events occurred in men < 75 years old, i.e., the largest age and gender subgroup. There was no notable difference in the number of patients experiencing fatal/life-threatening events between patients on invasive therapy versus those being medically managed. The proportion of patients experiencing major bleeding events (including fatal/life-threatening events) was similar between males and females (1.4 vs. 1.2%, respectively), and between patients receiving GPIIb/IIIa inhibitors versus those who were not (1.4 vs. 1.2%, respectively). Major bleeding events were reported in three patients aged ≥ 75 years (2.0%) versus in 24 patients aged < 75 years (1.3%), and in five patients who received medically managed therapy versus 22 receiving invasive therapy (2.0 vs. 1.3%, respectively).

### Non-bleeding AEs and SAEs

Of the safety population, 784 (39.2%) patients experienced at least one non-bleeding AE during treatment (Table S1 of Supplementary Material Online). The most common, non-bleeding events during treatment were hyperuricemia and dyspnea. Most patients reported non-bleeding AEs during treatment that were mild in intensity (28.4% of safety population), and only 4.0% of patients reported a severe event. During treatment, 39 (1.9%) patients discontinued study treatment due to a non-bleeding AE and 197 (9.8%) patients reported non-bleeding AEs that were considered to be treatment related by the local investigator.

Overall, 116 (5.8%) patients reported a non-bleeding SAE during treatment (Table S2 of Supplementary Material Online). The two most common, non-bleeding SAEs during treatment were chest discomfort (*n* = 6, 0.3%) and cardiac failure (*n* = 5, 0.2%). In total, 13 (0.6%) patients died due to a non-bleeding SAE, four (0.2%) discontinued study treatment due to a non-bleeding SAE, and 11 (0.5%) experienced events that were considered to be treatment related.

### AEs of Special Interest: Dyspnea and Hyperuricemia/Gout

Dyspnea AEs were reported in 68/2001 (3.4%) patients during ticagrelor treatment (Table S3 of Supplementary Material Online). Of these patients, 53 experienced mild dyspnea AEs. Dyspnea SAEs occurred in two patients during treatment and no patients died due to dyspnea. AEs related to hyperuricemia were reported in 130 (6.5%) patients during treatment, and gout was reported in eight (0.4%) patients (Table S3 of Supplementary Material Online). Most patients reported mild events during treatment (163, 8.1%). There were no reports of urate nephropathy. Three patients experienced hyperuricemia or gout SAEs during treatment and no event was fatal. Two patients discontinued the trial due to hyperuricemia/gout, and 86 patients experienced hyperuricemia or gout considered treatment-related by the investigator.

### AEs Related to Hepatic Function

During treatment, abnormal hepatic function AEs were recorded in 51/2001 (2.6%) patients (Table S3 of Supplementary Material Online). Of these patients, 44 reported a mild event, six reported a moderate event, and one reported a severe event. Most of these AEs were based on changes in aspartate aminotransferase (AST), alanine aminotransferase (ALT), and bilirubin, with liver function tests being higher than the upper limit of normal range at baseline; AST and ALT then decreased during the study. In the full-study cohort, mean (SD) AST levels decreased over time from 1.36 (2.01) μkat/L at visit 1 to 0.51 (3.87) μkat/L at the end of treatment, with a similar pattern for ALT. The severe AE was an SAE and was considered to be treatment-related by the investigator, thus, this patient discontinued the study. Overall, eight patients experienced treatment-related, abnormal hepatic function as judged by the investigator.

### Cardiovascular Events

**Fig. 2:**
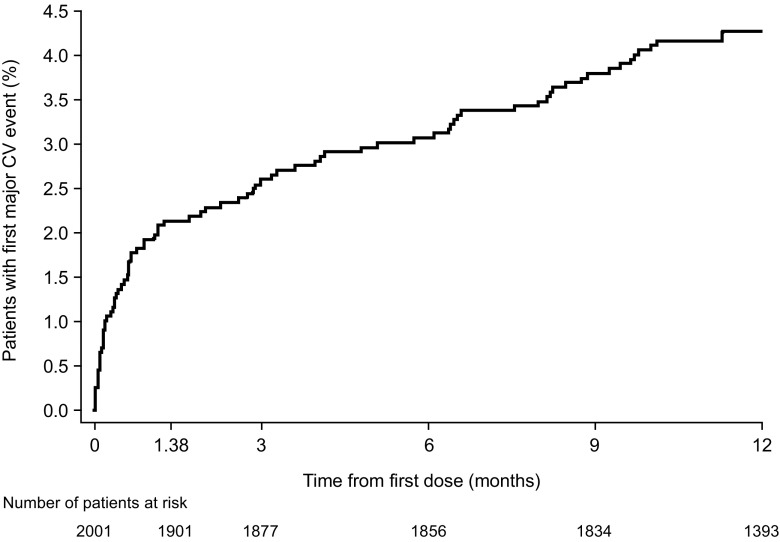
Kaplan-Meier plot of the first risk (cumulative incidence) of major cardiovascular event* (safety population). * a major cardiovascular (CV) event is defined as a composite of CV death, myocardial infarction, and stroke

In the safety population, 83 patients had a major CV event (i.e., composite of CV death, MI, or stroke) within 12 months after first exposure to ticagrelor, with a KM estimated event risk (95% CI) of 4.3% (3.5–5.3). Approximately 50% of the major CV events occurred during the first 6 weeks from the start of ticagrelor at the index event (Fig. 2). Of the safety population (KM estimated 12-month event risk [95% CI]), CV death occurred in 50 patients (2.6% [2.0–3.4]), and stroke occurred in 23 patients (1.2% [0.8–1.8]). A total of 21 MI events (NSTEMI *n* = 11, STEMI *n* = 8) occurred in 19 patients (KM estimated 12-month event risk [95% CI]: 0.9% [0.6–1.4]).

Of the 350 patients who discontinued ticagrelor before 12 months, a similar proportion of patients experienced major CV events (5.4%, 19/350) compared with the overall population (4.1%, 83/2001). For the 19 patients who discontinued ticagrelor and had a major CV event, 11 had the event while still on treatment and eight experienced the event after treatment discontinuation and within 12 months of study start.

For patients treated with invasive therapy (*n* = 1756), 66 patients had a major CV event within 12 months after first exposure to ticagrelor, KM estimated 12-month event risk (95% CI) was 3.8% (3.0–4.9). Of the 245 patients who were medically managed, 17 patients had a major CV event within 12 months after first exposure to ticagrelor, KM estimated 12-month event risk (95% CI) was 7.5% (4.7–11.8).

## Discussion

The DAYU study described the safety and rate of CV events during ticagrelor treatment in a large cohort of Chinese ACS patients, a population with limited data when ticagrelor was approved in China in 2012. The safety of ticagrelor in Chinese patients had been evaluated in major trials which included small numbers of Asian patients [14–16]. However, DAYU is the first study to describe clinical experience with ticagrelor in a larger group of Chinese ACS patients. The DAYU study demonstrated that ticagrelor 90 mg twice daily with low-dose aspirin for up to 1 year is associated with a low incidence of major bleeding events and SAEs, and a low incidence of major CV events in Chinese patients with ACS.

These findings are clinically important for several reasons. Firstly, as a phase IV study, the study population was aligned with the ticagrelor label in China and reflective of current clinical practice in this region (e.g., eligibility for ticagrelor treatment requires that patients with ACS must not have planned CABG within 7 days, must not have a history of intracranial bleeding, and must have no active bleeding). Secondly, the planned (12 months) and reported (309 days, ~ 10.2 months) mean duration of ticagrelor exposure in the DAYU study were aligned with label recommendations for ticagrelor in ACS patients [13] and international guidelines [5–9], and a high proportion of patients completed treatment. Additionally, demographic and baseline characteristics, medical history, and index event interventions in the DAYU study were similar to those observed in a real-world study in China (e.g., Chinese subgroup in the EPICOR Asia study [3]). In fact, the study population in the DAYU study was similar in characteristics, considering age, gender, STEMI frequency, and management strategy, to that included in the International EPICOR registry study [28].

Bleeding is the most common, clinically significant, safety concern during effective antiplatelet treatment, due to the mechanism of action [5–9, 29]. In the DAYU study, the incidence of PLATO-defined fatal/life-threatening bleeding and major bleeding in Chinese patients with ACS receiving ticagrelor was low (0.8 and 1.3%, respectively). Furthermore, the majority of bleeding events was minimal and did not require intervention. This pattern of bleeding events was consistent with previous ticagrelor data [14, 16, 30]. The low incidence of fatal bleedings (0.2%) in DAYU was consistent with those reported in the PLATO overall population (0.3%, 20/9235 [14]) and the PLATO Asian subpopulation (0.4%, 5/548 [16]), while reports of fatal/life-threatening bleedings were less frequent (0.8% [DAYU], 5.8% [PLATO overall] [14]). Furthermore, the incidence of major CV events was also lower in DAYU than observed with ticagrelor in PLATO [14], PLATO Asian substudy [16], and PHILO [30].

For non-bleeding events, the incidence of SAEs was generally low in the DAYU study. As expected in ACS patients, the most common non-bleeding SAEs were cardiac disorders. The two most commonly reported non-bleeding AEs were dyspnea and hyperuricemia, consistent with previous ticagrelor studies and the current ticagrelor prescribing information [13–15, 30, 31]. The incidence of dyspnea and hyperuricemia was low, and neither event was fatal. The dyspnea rate in DAYU (3.4%) was similar to that in a ticagrelor study in Japanese, Korean, and Taiwanese patients with ACS (5.7%; 22/401) [30], but lower than in the PLATO overall cohort (13.8%; 1270/9235) [14] or PLATO Asian subcohort [11.6%; 16]. The third most common AE in the DAYU study was abnormal hepatic function, which is not an AE associated with ticagrelor therapy [13]. In most cases, high levels of AST and ALT were recorded at baseline, which is expected after an ACS event. Elevated hepatic enzymes declined during continued ticagrelor treatment, which indicate a relationship to the background disease rather than ticagrelor. However, this observation highlights one key limitation in that the DAYU study was uncontrolled. Thus, the influence of confounding factors is more difficult to assess in the absence of a placebo arm. Of note, no patient in the DAYU study met the criteria for Hy’s Law at any time.

When contextualizing the above findings, important key differences between the DAYU and PLATO patient populations should be noted. Compared with PLATO patients, the DAYU population was generally younger, had fewer females, and exhibited fewer CV risk factors. In addition, PLATO and DAYU were conducted during different time periods, with PLATO starting in 2006 and DAYU commencing 7–8 years later, which may help explain why CV event rates are lower in the latter study due to management progression of these patients. Furthermore, as the DAYU study started after ticagrelor approval in China, recruited patients met the criteria of ACS patients described in the Chinese ticagrelor label, e.g., patients with planned CABG were excluded from DAYU, but were included in PLATO [14], and the described dyspnea side effect in the Chinese ticagrelor label may have impacted the recruitment of patients at risk for such symptoms. Exploratory analyses of the incidence of fatal/life-threatening bleeding and major bleeding in the DAYU study indicated that such events were generally consistent across the various patient subgroups assessed. However, it should be noted that the low incidence of bleeding in these categories and the imbalance in size of some subgroups make it difficult to draw firm, risk-related conclusions.

Overall, no new or unexpected safety findings were observed in the DAYU study, and the observed profile of ticagrelor reflected current Chinese prescribing information [13]. The safety profile with ticagrelor in Chinese patients with ACS was comparable to that reported in other large ticagrelor trials in a wide variety of patients [14, 15, 30, 32, 33].

There are several study limitations. DAYU was a single-arm, non-randomized study with no control arm, the design of which does not allow direct comparisons with other treatments. Hence, the results of the DAYU study were presented descriptively. However, this design was appropriate for the purpose of describing the safety of ticagrelor in a large population of contemporary Chinese patients experiencing an ACS event. An endpoint adjudication committee was not appointed to assess clinical outcomes in DAYU, which could be interpreted as a potential source of reporting bias. The potential risk of underreporting of AEs or CV events was mitigated by monitoring of participating sites. Although the DAYU study investigated the safety and incidences of CV events when used in a broad range of hospitals and in line with the Chinese prescribing information, it is still an interventional study where study drug was supplied to patients, which may have impacted treatment compliance. Patients with known or suspected contraindications to ticagrelor were excluded from the study, which may have resulted in a population with a lower risk of experiencing AEs commonly associated with this treatment (e.g., dyspnea) than in earlier studies. However, since the eligibility criteria were developed in line with the approved ticagrelor label, the DAYU study population should better reflect the patient population receiving ticagrelor in real life. Although a potential bias of this study is that patients were required to be enrolled as quickly as possible after presentation with ACS; therefore, it would not have been possible to enroll patients dying very early on presentation.

In conclusion, overall, the DAYU study demonstrated that exposure to ticagrelor plus aspirin for up to 1 year in a large population of Chinese patients with ACS resulted in a low rate of PLATO-defined major bleeding. The overall safety profile was in keeping with the current prescribing information and no new safety findings were found. Furthermore, the incidence of major CV events (CV death, MI, and stroke) was low during the study.

## Electronic supplementary material

ESM 1 (DOCX 25 kb)

## Abbreviations

ACS: acute coronary syndrome
AE: adverse event
ALT: alanine aminotransferase
AST: aspartate aminotransferase
b.i.d: twice daily
CABG: coronary artery bypass grafting
CFDA: Chinese Food and Drug Administration
CI: confidence interval
CV: cardiovascular
CVD: cardiovascular disease
DAPT: dual antiplatelet therapy
ECG: electrocardiogram
GPIIb/IIIa: glycoprotein IIb/glycoprotein IIIa
KM: Kaplan-Meier
MI: myocardial infarction
NSTEMI: non-ST elevation myocardial infarction
PCI: percutaneous coronary intervention
SAE: serious adverse event
SD: standard deviation
STEMI: ST elevation myocardial infarction
