# Supplementary material for: Safety and Incidence of Cardiovascular Events in Chinese Patients with Acute Coronary Syndrome Treated with Ticagrelor: the 12-Month, Phase IV, Multicenter, Single-Arm DAYU Study
Source: Cardiovasc Drugs Ther. 2018 Feb 28;32(1):47–56. doi: 10.1007/s10557-018-6772-3 (PMC5843700; doi:10.1007/s10557-018-6772-3)
Supplement: Supplementary file 1 — (DOCX 25 kb) [file 10557_2018_6772_MOESM1_ESM.docx]

**Electronic Supplementary Material**

**Safety and Incidence of Cardiovascular Events in Chinese Patients with Acute Coronary Syndrome Treated with Ticagrelor: The 12-month, Phase IV, Multicenter, Single-arm DAYU Study**

**Runlin Gao^*^• Yongjian Wu^1^• Hengliang Liu^2^• Guohai Su^3^• Zuyi Yuan^4^• Aidong Zhang^5^• Yong Wang^6^• Zhirong Wang^7^• Yan Wang^8^• Huanyi Zhang^9^• Yang Zheng^10^• Lei Liu^11^• Lijun Shen^11^• Maria Leonsson-Zachrisson^12^• Yaling Han^13^• on behalf of the DAYU study investigators**

*****Corresponding author at Fuwai Hospital, National Center for Cardiovascular diseases, Chinese Academy of Medical Sciences, 167 Beilishi Road, Xi Cheng District, Beijing, China. E-mail: [gaorunlin@citmd.com](mailto:gaorunlin@citmd.com)

**Supplementary methods**

**Definition of PLATO-defined bleeding events**

Major fatal/life-threatening bleeding was defined as fatal, intracranial, or intrapericardial bleeding with cardiac tamponade, hypovolemic shock or severe hypotension due to bleeding and requiring pressors or surgery, clinically overt or apparent bleeding associated with a decrease in hemoglobin of >50 g/L, or need for transfusion of ≥4 units of red blood cells (RBCs). Other major bleeding was defined as events leading to significant disability (e.g. intraocular with permanent vision loss), clinically overt or apparent bleeding associated with a decrease in hemoglobin of 30–50 g/L, or need for transfusion of 2–3 units of RBCs. Minor bleeding was defined as any event requiring medical intervention and minimal bleeding was defined as events not requiring treatment (e.g. bruising, bleeding gums, etc.).

**Participating sites (all in China)**

| Site no. | Site name |
| --- | --- |
| 0001 | Fuwai Hospital, Chinese Academy of Medical Sciences |
| 0003 | General Hospital of the Chinese people's Liberation Army |
| 0004 | Peking University Third Hospital |
| 0005 | Beijing Union Medical College Hospital |
| 0006 | The Beijing Friendship Hospital, Capital Medical University |
| 0008 | Beijing Hospital |
| 0009 | Beijing China-Japan Friendship Hospital |
| 0010 | Xiamen City Heart Center |
| 0011 | Affiliated Union Hospital of Fujian Medical University |
| 0012 | Fujian Provincial Hospital |
| 0014 | First affiliated hospital of Sun Yat-sen University |
| 0016 | People's Hospital of Shenzhen |
| 0017 | Sun Yat-Sen Cardiovascular Hospital |
| 0018 | People's Hospital of Zhongshan City |
| 0020 | Guangxi Zhuang Autonomous Region People's Hospital |
| 0021 | The First Hospital of Jilin University |
| 0022 | People's Hospital of Henan province |
| 0023 | People's Hospital of Zhengzhou |
| 0024 | The Second Hospital of Jilin University |
| 0025 | Wuhan Asia Heart Hospital |
| 0026 | People's Hospital of Jiangsu Province |
| 0028 | Big hospital, Southeast University |
| 0029 | General Hospital of Shenyang Military Area Command |
| 0030 | People's Hospital of Liaoning Province |
| 0031 | The Second Hospital of Shandong University |
| 0032 | Qilu Hospital of Shandong University |
| 0033 | First Affiliated Hospital, The Fourth Military Medical University |
| 0034 | XI an Jiaotong University, The First Hospital |
| 0035 | Shanghai Changhai Hospital |
| 0036 | Shanghai Ruijin Hospital |
| 0038 | Huashan Hospital Affiliated to Fudan University |
| 0041 | Shanxi Provincial Cardiovascular Hospital |
| 0042 | First people's Hospital in Jining city |
| 0043 | Kunming, Chengdu Military Region General Hospital |
| 0045 | Shandong Provincial Hospital |
| 0047 | Xuzhou Medical College Affiliated Hospital |
| 0050 | Fourth Affiliated Hospital of China Medical University |
| 0051 | Hospitals of Zhejiang Province |
| 0052 | Huaxi Hospital of Sichuan University |
| 0053 | People's Hospital of Wuxi |
| 0056 | Beijing Tongren Hospital Affiliated to Capital Medical University |
| 0060 | The Third Affiliated Hospital of Sun Yat-sen University |
| 0061 | Shenzhen Hospital of Beijing University |
| 0062 | The Third Hospital of Hebei Medical University |
| 0063 | Hebei Province People's Hospital |
| 0065 | Second Affiliated Hospital of Harbin Medical University |
| 0066 | CR of WISCO General Hospital |
| 0067 | Daqing Oilfield Head Hospital |
| 0068 | Tangshan Worker's Hospital |
| 0071 | Shengjing Hospital (The Second Affiliated Hospital of China Medical University) |
| 0072 | Taian City hospital |
| 0074 | Shanghai Tenth People's Hospital |
| 0076 | Shanghai Changzheng Hospital |
| 0077 | Shanghai Oriental Hospital |
| 0080 | Tianjin Hospitals |
| 0081 | General Hospital of Tianjin Medical University |
| 0082 | First affiliated Hospital of Zhejiang University School of medicine |
| 0083 | Sir Run Shaw Hospital Affiliated to Zhejiang University School of Medicine |
| 0084 | Taizhou Hospital, Zhejiang Province |
| 0085 | First Hospital of Ningbo City |
| 0086 | Zhejiang Hospital |
| 0088 | Jiangyin City People's Hospital |
| 0089 | The Second Affiliated Hospital of Suzhou University |
| 0090 | Jinan Central Hospital |
| 0091 | Guangzhou Red Cross Hospital |
| 0092 | First Affiliated Hospital of Jinan University |
| 0093 | Panyu Central Hospital |
| 0094 | Xiangya Hospital |
| 0095 | Second Xiangya Hospital |
| 0096 | Third Xiangya Hospital |
| 0097 | Jiangsu Province Hospital of Traditional Chinese Medicine |
| 0098 | The Xiangtan Central Hospital |
| 0100 | Xinjiang Uygur Autonomous Region People's Hospital |
| 0101 | First Affiliated Hospital of Xinjiang Medical University |
| 0102 | Traditional Medical Hospital of the Xinjiang Uygur Autonomous Region |
| 0103 | First Hospital of Nanjing |
| 0105 | First Hospital Affiliated to Nanhua University |
| 0106 | Third people's Hospital of Hubei Province |
| 0107 | Peking University Shougang Hospital |
| 0108 | Hebei Bethune International Peace Hospital |
| 0109 | Yantai Yuhuangding Hospital |
| 0110 | Central Hospital of Tongji Medical College of Hust in Wuhan |
| 0112 | First Affiliated Hospital of Xiamen University |
| 0115 | First Affiliated Hospital of Dalian Medical University |
| 0116 | Central Hospital of Tongji Medical College of Hust in Jingzhou |
| 0117 | Binzhou Medical College Hospital Cardiology |
| 0118 | Hunan Provincial People's Hospital |
| 0119 | Beijing General Hospital of Second Artillery Corps |
| 0120 | People's Hospital of Foshan City, First |
| 0121 | Sun Yat-Sen Sun Yat-sen Memorial Hospital |
| 0122 | Second Affiliated Hospital of Dalian Medical University |
| 0123 | Nineth people's Hospital of Shanghai Jiaotong University School of Medicine |
| 0124 | The Second Hospital of Shanxi Medical University |
| 0125 | Wuxi Second people's Hospital |
| 0127 | Sino-Japanese Friendship Hospital of Jilin University |
| 0128 | The Second Hospital of Hebei Medical University |
| 0130 | General Hospital of Yankuang Group |
| 0133 | Weihai Municipal Hospital |
| 0135 | PuTuo District, Shanghai City Center Hospital |
| 0136 | Shanghai Tongren Hospital |
| 0137 | Qingdao Haici Medical Group |
| 0138 | Qingdao Municipal Hospital |
| 0139 | Fourth Affiliated Hospital of Harbin Medical University |
| 0140 | Dalian Central Hospital |

**Table S1:** Non-bleeding AEs (overall incidence ≥1%) by system organ class and preferred term (safety population)

| System organ class  preferred term | Ticagrelor 90 mg b.i.d  *n* = 2001 |
| --- | --- |
|  | During treatment  *n* (%) |
| Patients with at least one event | 784 (39.2) |
| Metabolism and nutrition disorders | 201 (10.0) |
| Hyperuricemia | 130 (6.5) |
| Hyperlipidemia | 31 (1.5) |
| Infections and infestations | 155 (7.7) |
| Upper respiratory tract infection | 50 (2.5) |
| Pneumonia | 26 (1.3) |
| Lung infection | 21 (1.0) |
| Respiratory, thoracic and mediastinal disorders | 139 (6.9) |
| Dyspnea | 68 (3.4) |
| Cough | 42 (2.1) |
| Investigations | 113 (5.6) |
| Increased blood uric acid | 46 (2.3) |
| General disorders and administration site conditions | 99 (4.9) |
| Chest discomfort | 35 (1.7) |
| Pyrexia | 29 (1.4) |
| Cardiac disorders | 82 (4.1) |
| Hepatobiliary disorders | 71 (3.5) |
| Abnormal hepatic function | 51 (2.5) |
| Nervous system disorders | 50 (2.5) |
| Dizziness | 25 (1.2) |
| Renal and urinary disorders | 46 (2.3) |
| Skin and subcutaneous tissue disorders | 42 (2.1) |
| Blood and lymphatic system disorders | 37 (1.8) |
| Anemia | 26 (1.3) |
| Vascular disorders | 22 (1.1) |

AE, adverse events; b.i.d, twice daily

**Table S2:** Non-bleeding SAEs (overall incidence ≥0.1%) by system organ class and preferred term (safety population)

| System organ class  preferred term | Ticagrelor 90 mg b.i.d  *n* = 2001 |
| --- | --- |
|  | During treatment  *n* (%) |
| Patients with at least one event | 116 (5.8) |
| Cardiac disorders | 25 (1.2) |
| Cardiac failure | 5 (0.2) |
| Acute left ventricular failure | 3 (0.1) |
| Chronic cardiac failure | 3 (0.1) |
| Infections and infestations | 19 (0.9) |
| Lung infection | 4 (0.2) |
| Pneumonia | 3 (0.1) |
| Appendicitis | 2 (0.1) |
| Urinary tract infection | 2 (0.1) |
| General disorders and administration site conditions | 10 (0.5) |
| Chest discomfort | 6 (0.3) |
| Sudden death | 3 (0.1) |
| Musculoskeletal and connective tissue disorders | 8 (0.4) |
| Intervertebral disc protrusion | 4 (0.2) |
| Gouty arthritis | 2 (0.1) |
| Renal and urinary disorders | 8 (0.4) |
| Chronic kidney disease | 4 (0.2) |
| Gastrointestinal disorders | 7 (0.3) |
| Gastritis | 4 (0.2) |
| Metabolism and nutrition disorders | 7 (0.3) |
| Inadequate control of diabetes mellitus | 4 (0.2) |
| Type 2 diabetes mellitus | 2 (0.1) |
| Respiratory, thoracic and mediastinal disorders | 7 (0.3) |
| Chronic obstructive pulmonary disease | 2 (0.1) |
| Dyspnea | 2 (0.1) |
| Injury, poisoning and procedural complications | 6 (0.3) |
| Road traffic accident | 2 (0.1) |
| Vascular disorders | 6 (0.3) |
| Neoplasms benign, malignant and unspecified (including cysts and polyps) | 5 (0.2) |
| Malignant lung neoplasm | 2 (0.1) |
| Nervous system disorders | 5 (0.2) |
| Hepatobiliary disorders | 4 (0.2) |
| Psychiatric disorders | 3 (0.1) |
| Depression | 2 (0.1) |
| Blood and lymphatic disorders | 2 (0.1) |
| Ear and labyrinth disorders | 2 (0.1) |
| Endocrine disorders | 2 (0.1) |
| Eye disorders | 2 (0.1) |
| Reproductive system and breast disorders | 2 (0.1) |

b.i.d, twice daily; SAE, serious adverse event

**Table S3:** AEs of special interest during treatment by system organ class and preferred term (safety population)

| System organ class  preferred term | Ticagrelor 90 mg b.i.d (*n* = 2001) |
| --- | --- |
|  | During treatment  *n* (%) |
| Respiratory, thoracic and mediastinal disorders  Dyspnea  Patients with at least 1 AE  Patients with at least 1 SAE  Leading to study drug discontinuation  Related to study drug | 68 (3.4)  68 (3.4)  68 (3.4)  2 (0.1)  13 (0.6)  37 (1.8) |
| Metabolism and nutrition disorders  Hyperuricemia  Gout  Investigations  Increased blood uric acid  Musculoskeletal and connective tissue disorders  Gouty arthritis  Patients with at least 1 AE  Patients with at least 1 SAE  Leading to study drug discontinuation  Related to study drug | 135 (6.7)  130 (6.5)  8 (0.4)  46 (2.3)  46 (2.3)  3 (0.1)  3 (0.1)  180 (9.0)  3 (0.1)  2 (0.1)  86 (4.3) |

AE, averse event; b.i.d, twice daily; SAE, serious adverse event
